# Presence of Protozoan Viruses in Vaginal Samples from Pregnant Women and Their Association with Trichomoniasis

**DOI:** 10.3390/pathogens14080764

**Published:** 2025-08-01

**Authors:** Gegham Ghardyan, Lusine Abrahamyan, Karen Julhakyan, Hakob Davtyan, Norayr Martirosyan, Elina Arakelova, Hranush Avagyan, Sona Hakobyan, Tigranuhi Vardanyan, Naira Karalyan, Zaven Karalyan

**Affiliations:** 1 Department of Obstetrics and Gynecology N 2, Yerevan State Medical University, Yerevan 0093, Armenia; 2 Republican Institute of Reproductive Health, Perinatology, Obstetrics and Gynaecology, Yerevan 0078, Armenia; 3 Obstetrics Department, St. Gregory the Illuminator Medical Center, Yerevan 0056, Armenia; 4 Department of Obstetrics and Gynecology of National Institute of Health After Avdalbekyan, Yerevan 0051, Armenia; 5 CANDLE Synchrotron Research Institute, 31 Acharyan Str, Yerevan 0022, Armenia; 6 Laboratory of Cell Biology and Virology, Institute of Molecular Biology of NAS RA, Yerevan 0014, Armenia; 7 Experimnetal Laboratory, Yerevan State Medical University, Yerevan 0093, Armenia; 8 Scientific Center for Risk Assessment and Analysis in Food Safety Area, 107/2 Masis Highway, Yerevan 0071, Armenia; 9 Department of Pathological Anatomy and Clinical Pathology, Yerevan State Medical University, Yerevan 0093, Armenia

**Keywords:** *Trichomonas vaginalis* (TV), *Trichomonas vaginalis* virus (TVV), giant protozoan viruses, pregnant women, preterm birth, qRT-PCR, pathogenesis, reproductive health

## Abstract

This study was conducted in Armenia and included 32 pregnant women with TV infection and 30 healthy controls. The vaginal virome includes viruses that infect human cells and unicellular eukaryotes such as *Trichomonas vaginalis* (TV). Among these are *Trichomonas vaginalis* viruses (TVVs), double-stranded RNA viruses from the *Totiviridae* family, and giant DNA viruses that replicate in protozoa. This study investigated the presence of TVVs and giant protozoan viruses in pregnant women with trichomoniasis in Armenia and explored their potential associations with adverse pregnancy outcomes. Vaginal and urethral samples were collected from 32 pregnant women with confirmed TV infection and 30 healthy pregnant controls. TVVs and giant viruses (*Marseilleviridae*, *Mimiviridae*, *Phycodnaviridae*) were detected using qRT-PCR. Viral RNA and DNA were extracted from clinical samples and TV cultures, followed by quantification and gene expression analysis. Selected TVVs were visualized via scanning electron microscopy. All TV-positive women carried at least one TVV strain, with 94% harboring multiple TVV types and TVV4 being the most common. TV infection was significantly associated with preterm birth and premature rupture of membranes (PPROM). Giant viruses were identified in all TV-positive cases but in only 40% of controls. *Marseilleviridae* gene expression was observed in TV cultures, suggesting possible interactions. These findings highlight a potential role for protozoan viruses in reproductive complications and warrant further investigation.

## 1. Introduction

### 1.1. Vaginal Virome

The vaginal virome comprises a diverse array of viruses present within the female genital tract, including both eukaryotic viruses that infect human cells and bacteriophages that target bacterial populations. These viral communities play a critical role in shaping reproductive and sexual health through complex interactions with the vaginal microbiota and the host immune system [1]. While bacteriophages within the vaginal virome have been relatively well characterized, eukaryotic viruses remain substantially understudied. Nonetheless, several families of eukaryotic viruses, such as *Herpesviridae* and *Papillomaviridae,* have been detected in vaginal samples, suggesting potential roles in modulating local immune responses and influencing microbial community structure [2]. Beyond traditional viral classifications, eukaryotic viruses in the vaginal environment can be further distinguished by their host specificity for those infecting human cells and those targeting unicellular eukaryotic organisms, such as protozoa. Recent research has revealed increasing complexity within the vaginal virome, particularly with the identification of virus-like particles (VLPs) and true viruses associated with the protozoan parasites that themselves infect humans. The expanding use of high-throughput sequencing technologies is expected to uncover many more of these virus–protozoa associations. Notably, viruses that infect *Trichomonas vaginalis* (TV) have been identified, alongside viruses associated with other protozoa, including amoebae and unicellular algae.

### 1.2. Trichomonas Vaginalis and TVVs

Urogenital trichomoniasis, caused by the flagellated protozoan *TV*, is the most common non-viral sexually transmitted infection (STI) worldwide, affecting an estimated 2–10% of the global population [3]. In women, it is associated with vaginitis, cervicitis, pelvic inflammatory disease, infertility, and adverse pregnancy outcomes [4,5,6]. Its clinical impact varies depending on host immunity and parasite strain diversity. Trichomoniasis also increases the risk of HIV acquisition and often occurs alongside other STIs [7,8].

A recently recognized aspect of TV infection is its symbiosis with *Trichomonas vaginalis virus* (TVV), a double-stranded RNA virus of the *Totiviridae* family. Multiple subtypes (TVV1–TVV5) have been identified [9,10,11], and studies suggest these viruses modulate the gene expression and virulence of TV [12,13]. However, the role of TVVs in pregnancy-related complications remains unclear. Adding further complexity, viruses associated with TV or its viral symbionts may include viruses of mammalian origin, though their roles remain unexplored.

### 1.3. Giant Protozoan Viruses in Human Samples

Similarly, members of the nucleocytoplasmic large DNA virus (NCLDV) group—best known for infecting free-living amoebae such as *Acanthamoeba* spp.—have recently been detected in the vaginal virome [14]. While they are not known to infect humans directly, their presence in human-associated environments raises questions about their potential role in modulating host–microbe interactions or serving as reservoirs of virulence genes.

This study aims to investigate the presence and diversity of *Trichomonas vaginalis* viruses (TVVs) and other eukaryotic viruses, including members of the nucleocytoplasmic large DNA virus (NCLDV) group, in vaginal samples from pregnant women in the Republic of Armenia. We specifically seek to determine whether these viruses, either directly or through interactions with their protozoan hosts such as *T. vaginalis*, are associated with adverse reproductive health outcomes. Our objective is to better understand the potential virome–protozoa–host interactions that may influence maternal health and pregnancy.

## 2. Materials and Methods

### 2.1. Participants and Ethical Approval

Participants gave written informed consent for participation in the study and the use of stored isolates. The study protocol was approved by the Republican Institute of Reproductive Health, Perinatology, Obstetrics and Gynaecology (Yerevan, Armenia). Laboratory personnel had no contact with participants and no access to personal identifiers.

Inclusion criteria: Pregnant women aged 18–45 years; gestational age between 8 and 35 weeks at the time of sample collection; attendance at the Republican Institute of Reproductive Health, Perinatology, Obstetrics and Gynecology in Yerevan, Armenia; and provision of written informed consent for participation and sample use. For the case group, the inclusion criteria were as follows: Confirmed diagnosis of TV infection by either wet mount microscopy or culture. For the control group, the inclusion criteria included the following: Negative for TV infection by both wet mount microscopy and culture.

Exclusion criteria: Presence of other diagnosed sexually transmitted infections (e.g., *Chlamydia trachomatis*, *Neisseria gonorrhoeae*, *Mycoplasma* spp.) at the time of sampling; use of systemic or vaginal antibiotics, antifungals, or antivirals within the past 4 weeks; evidence of active vaginal bleeding or recent gynecologic procedures (<2 weeks); multiple pregnancies (e.g., twins, triplets); known immunodeficiency disorders or current immunosuppressive therapy; and inability or unwillingness to provide informed consent.

### 2.2. Sample Collection

This study employed a case–control design. The case group consisted of pregnant women (*n* = 32) diagnosed with TV infection, either symptomatic or asymptomatic, attending the Republican Institute of Reproductive Health, Perinatology, Obstetrics and Gynecology in Yerevan, Armenia, between October 2023 and May 2024. The control group comprised pregnant women without evidence of trichomoniasis (*n* = 30), confirmed by both microscopic examination and culture, and matched by gestational age and clinical setting. Vaginal and urethral swabs were collected from all participants. In the case group, additional isolates were used for viral analysis and cultivation. Swabs were collected using sterile saline-pre-moistened cotton swabs and processed for both direct diagnostic testing and further virological evaluation.

### 2.3. Diagnostic Testing (TV Detection)

Two diagnostic methods were applied to detect TV in vaginal swabs: (1) routine wet mount microscopy to identify motile protozoa, and (2) inoculation into Diamond’s culture medium (Hardy Diagnostics, Santa Maria, CA, USA) for confirmation and isolation [15]. Only participants with positive results from either method were included in the case group. Controls tested negative on both assays.

### 2.4. Viral Nucleic Acid Extraction

Viral RNA/DNA was extracted using the HiGene™ Prep kit (BIOFACT, Daejeon, Republic of Korea) and reverse transcribed with the FIREScript^®^ RT cDNA kit (Solis Biodyne, Tartu, Estonia), following manufacturer protocols. Concentrations and A260/280 ratios (~1.8 for DNA, ~2.0 for RNA) were measured using a NanoDrop^®^ ND-1000 spectrophotometer (Thermo Fisher Scientific, Wilmington, DE, USA) [16].

### 2.5. Detection of TVVs and Giant Viruses

TVVs was assessed by quantitative reverse transcription PCR (qRT-PCR) using complementary DNA (cDNA) synthesized from RNA extracted from vaginal secretions and primary TV cultures. TVVs were detected in both sample types. For qRT-PCR, equivalent volumes of fluid were used from vaginal samples and culture media to ensure consistency in viral detection.

In parallel, giant viruses belonging to the families Marseilleviridae, Mimiviridae, and Phycodnaviridae were detected using qRT-PCR performed on extracted DNA samples. These viruses were also identified in both vaginal secretions and primary TV cultures. As with TVV analysis, equal volumes of material were processed for each sample type to maintain methodological consistency. The sensitivity of all primers was confirmed via serial dilutions and melt curve analysis. All primers amplified their respective targets with high efficiency and were capable of detecting viral templates at concentrations as low as 10^2^–10^3^ copies per reaction. Negative (no-template) and positive controls were included in each reaction to monitor for contamination and validate assay performance.

qRT-PCR was performed using the standard curves and the SYBR green method previously described [17,18] on a Bio-Rad CFX 96 Real-Time PCR system (Bio-Rad Laboratories, Inc. Hercules, CA, USA). To quantify viral RNA, a standard with a known quantity of genome copies African swine fever virus (ASFV) is used. In this study, a culture of porcine alveolar macrophages (PAM) infected with ASFV was utilized. Standard curves were generated by serial 10-fold dilutions of viral DNA [14,17]. The fluorescence threshold value (Ct) was determined using CFX Maestro Software version 2.3 (Bio-Rad Laboratories, Inc., Hercules, CA, USA). Analysis of gene expression of giant viruses by quantitative real-time PCR was performed using previously described [19].

Each 20 µL qPCR reaction contained 4 µL of 5× HOT FIREPol^®^ EvaGreen^®^ Mix (ROX), 0.3 µL of each primer (100 pmol/µL), 4 µL of DNA/cDNA template, and 11.6 µL of ddH_2_O. DNA from ASFV-infected pig’s spleen served as a positive control, with ddH_2_O as the negative control. Reactions were run using CFX Maestro Software (Bio-Rad Laboratories, Hercules, CA, USA) under the following conditions: 95 °C for 12 min, followed by 40 cycles of 95 °C for 15 s, 52 °C for 30 s, and 72 °C for 30 s for Melting curve. Primers for ASFV and TVV1–4 were selected from previously published, peer-reviewed literature [11,18,19]. Additionally, primer design was performed for TVV5 and for giant viruses representing three families: *Mimiviridae, Phycodnaviridae*, and *Marseilleviridae*. All sequences were obtained in FASTA format and primers were ordered from Integrated DNA Technologies (IDT) (https://eu.idtdna.com/pages, accessed on 11 May 2019). For alignment of the cDNA plots and infection titers of ASFV, Cq values were rescaled after comparing with viral genome copies and modified in absolute amounts along the *y*-axis for better visualization. All primers and sequence GenBank IDs used in the experiments are presented in Supplementary Table S1.

### 2.6. SEM Imaging

The culture medium containing TV was subjected to two freeze–thaw cycles. The resulting lysates were centrifuged at 3000× *g* for 10 min at room temperature (~25 °C) to remove cellular debris. The supernatants were then homogenized using a Virtis 45 Tissue Homogenizer (815 State Route 208, Gardiner, NY, 12525, USA) at 45,000 rpm for 3 min. The homogenized samples were resuspended in double-distilled water and subsequently centrifuged at 25,000 rpm for 5 min at 4 °C, without the use of a density gradient [20].

The sputter-coated samples obtained from above-mentioned procedures were subjected to high-resolution electron imaging using Carl ZEISS SEM EVO 10 (Carl Zeiss AG Vertriebs GmbH, Düsseldorf, Germany), scanning electron microscopes were used to reveal the virus particles in the centrifugated lysates.

### 2.7. Statistical Analysis

All in vitro virus analyses were performed in triplicate. Statistical analysis was conducted using SPSS version 17.0 (SPSS Inc., Chicago, IL, USA) and GraphPad Prism version XX (Prism 10.4.2). For comparisons of continuous variables, either the two-tailed Student’s *t*-test or Mann–Whitney U test was used depending on data distribution. Categorical variables, including pregnancy outcomes, were assessed using the Chi-square test or Fisher’s exact test where appropriate. A Chi-square test applied to the full dataset of pregnancy outcomes yielded a value of 152.8 (df = 5, critical value = 11.07, *p* < 0.001). Individual outcomes (e.g., preterm birth and PPROM) were analyzed using 2 × 2 contingency tables; for example, the association between trichomoniasis and preterm birth yielded a Chi-square value of 148.6 (df = 1, *p* < 0.001), indicating a statistically significant difference compared with the background population rate (2.3%). A post hoc power analysis using G*Power 3.1 confirmed that the sample size (n = 62; 32 TV-positive cases and 30 controls) provided 80% power to detect differences in categorical outcomes with an alpha of 0.05. A *p*-value < 0.05 was considered statistically significant for all tests.

## 3. Results

### 3.1. Association Between TV Infection and Pregnancy Complications

The study was conducted between October 2023 and May 2024, during which 3476 pregnancies were registered (Republican Institute of Reproductive Health, Perinatology, Obstetrics and Gynaecology, Yerevan, Armenia). Of these, trichomoniasis was diagnosed in 32 pregnant women. Data related to pregnancy outcomes and trichomoniasis are presented in Supplementary Table S2.

The majority of the 32 women included in the study (n = 30) were symptomatic, only 2 had no complaints. The main complaints of the pregnant women we observed were itching and vaginal discharge. These complaints correspond to the most common complaints reported in the international literature [4,6]. We investigated the occurrence of symptoms reported by patients, laboratory indices in those infected with TV, and the presence of various TVVs. Among the 32 women with TV infection, 12 (37.5%) experienced preterm birth and/or preterm premature rupture of membranes (PPROM), compared with 83 cases out of 3476 pregnancies (2.3%) in the general obstetric population. A Chi-square test comparing these proportions yielded a value of 148.6 (df = 1, *p* < 0.001). Notably, 10 of the 12 TV-positive women with preterm birth were also positive for at least one subtype of *Trichomonas vaginalis* virus (TVV1–4). Preterm birth was assessed based on gestational age, clinical signs, and cervical length measurements. PPROM was diagnosed using clinical presentation, speculum examination, microscopic evaluation of vaginal smears, and the Amniotest. These findings suggest a potential association between TVV carriage and adverse pregnancy outcomes (Supplementary Table S3)

No significant correlation was identified between the presence or absence of the studied symptoms and the presence of TVVs or their combinations.

As no cases of TV infection without concurrent TVVs or giant viruses were observed in our study population, we are unable to determine whether the observed pathology is attributable to the protozoan itself or its association with viral elements. Nonetheless, the association between the pathology and the presence of both the protozoan and the investigated viruses appears evident.

### 3.2. Prevalence of TVV Subtypes in Infected Patients

**Figure 1:**
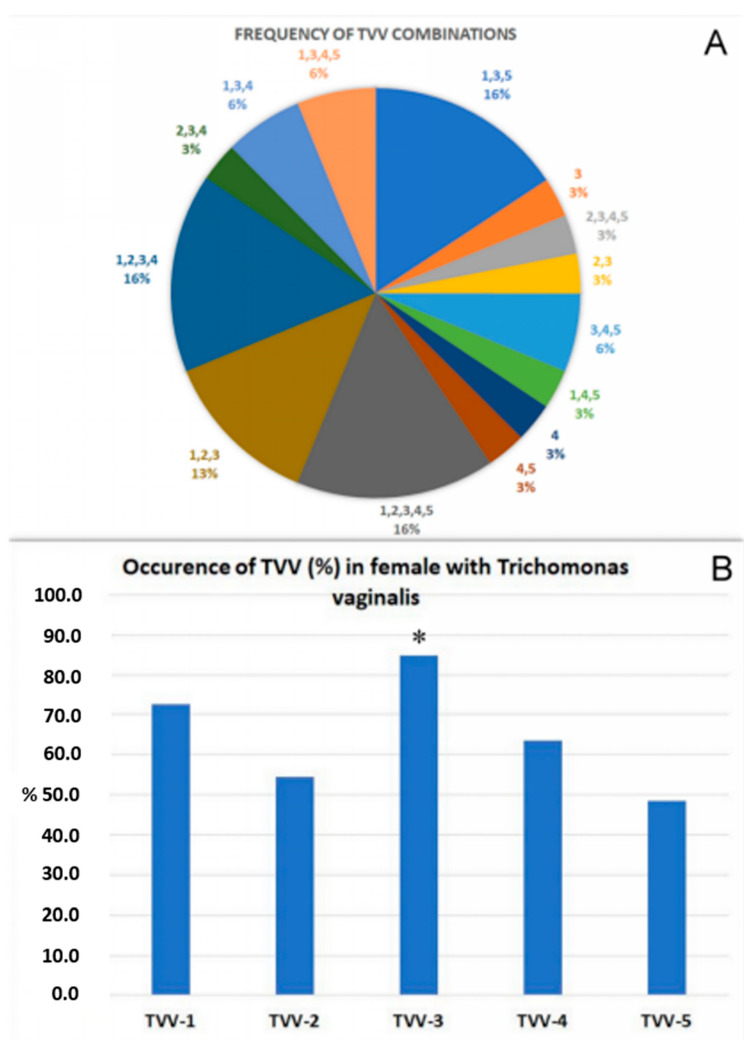
(**A**) Frequency of TVV combinations in pregnant female with trichomoniasis. (**B**) Occurrences of TVV strains in pregnant female with trichomoniasis * Significant compared with TVV-3 and TVV-5 (*p* < 0.05).

In this work, 32 samples of TV isolated in Armenia were analyzed for the presence of the five species of TVV (TVV1-5). Analysis of the data revealed the presence of TVV in all patients with TV. Most women with TV have a combination of 2 or more TVVs (94%). All 5 strains of TVV occur in 14% of patients (Figure 1A). When analyzing the distribution of viruses, it was shown that the most common virus is TVV-3, and the least common are TVV-2 and TVV-5 (Figure 1B). The presence of only one virus of the TV was determined at 3 and 4 TVV and was quite rare (3% each).

### 3.3. Quantitative Analysis of TVV RNA Levels in Vaginal Swabs and T. vaginalis Cultures

**Figure 2:**
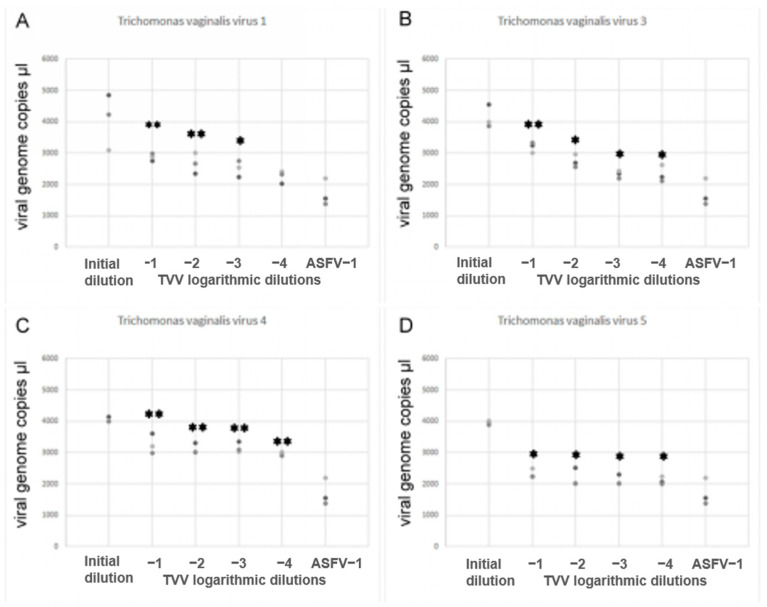
Quantification of the TVV RNA of vaginal smear samples (on TVV-2 example). * Significant standard curves for the quantification of ASFV DNA levels were compared with TVV RNA levels (*p* < 0.05). (**A**) isolate 1, (**B**) isolate 3, (**C**) isolate 4, and (**D**) isolate 5. ** significantly low compared to-1, -2, -3 dilutions.

**Figure 3:**
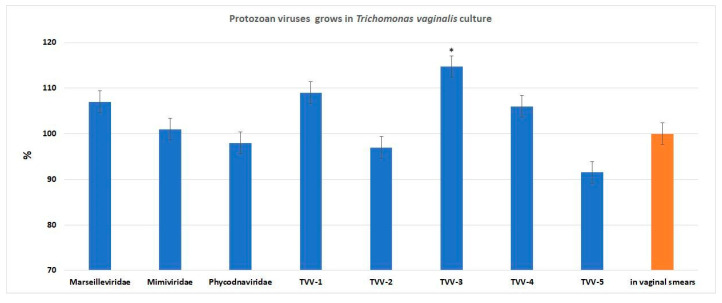
Quantification of the giant virus DNA and TVV RNA in TV culture.

To demonstrate the results, a random sample containing four TVV isolates was selected: isolate 1 (Figure 2A), isolate 3 (Figure 2B), isolate 4 (Figure 2C), and isolate 5 (Figure 2D). Figure 3 illustrates the RNA quantification of TVVs by qRT-PCR assay in vaginal smear samples.

As shown in Figure 2, qRT-PCR reliably detects a quantitative difference between the initial dilution and the first logarithmic dilution. The difference in subsequent logarithmic dilutions is less pronounced but still generally observable. By comparing the gene copies in log^−1^ dilution of the African swine fever (ASF) virus with reference, an approximate estimation of the TVV genome copy number can be made. Additionally, the quantitative changes in TVV types were assessed in the Diamond medium used for TV propagation. A quantitative increase in TVV type 3 was observed when compared with the viral load in vaginal smears (Figure 3).

Figure 3 presents the viral loads measured by quantitative PCR, expressed as genome copies/mL. Viral DNA and RNA levels in vaginal samples were set as the baseline (100%, control), and the figure shows the arithmetic mean percentage change of these viral loads in trichomonad cultures derived from the same samples across all patients. * Significant viral DNA and RNA quantification occurred in culture when compared with control viral DNA and RNA level (*p* < 0.05)

### 3.4. Electron Microscopy Visualization of TVV Particles in TV Cultures

Visualization of TVV is most convenient when isolating viruses from TV culture samples. As the culture contains significantly fewer foreign (non-TVV) viral particles, compared with vaginal samples.

**Figure 4:**
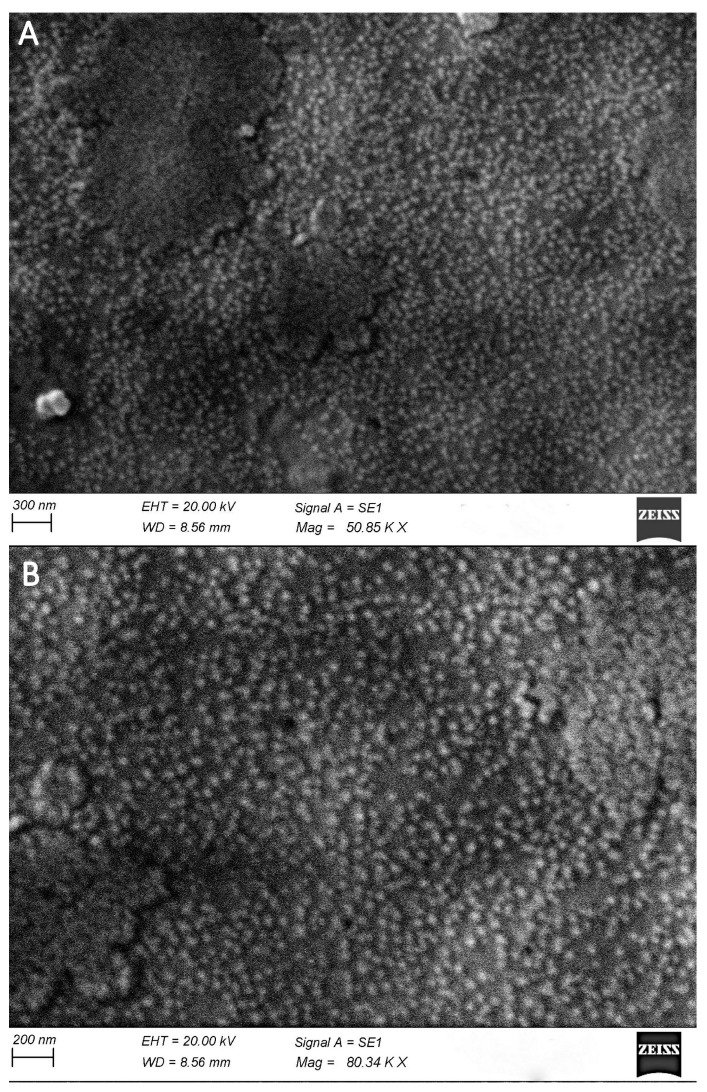
Scanning electron microscopy (SEM) images showing TVV-like particles. (**A**). Surface view at 50,850× magnification reveals dense clusters of spherical particles, consistent with viral morphology, distributed across the mite cuticle. (**B**). The higher magnification image (80,340×) highlights the uniform size and distribution of these particles, suggestive of a potential viral infection or accumulation. Both images were acquired using a Zeiss SEM with an accelerating voltage (EHT) of 20.00 kV and a working distance (WD) of 8.56 mm. Signal A = SE1.

Figure 4A,B shows the images of TVV isolated from primary lysates of the TV culture at different magnifications, 48 h after the start of cultivation. Our data were the first to visualize TVV using SEM from TV culture samples.

### 3.5. Detection of Giant DNA Viruses in Vaginal Samples from TV-Infected and Uninfected Women

**Figure 5:**
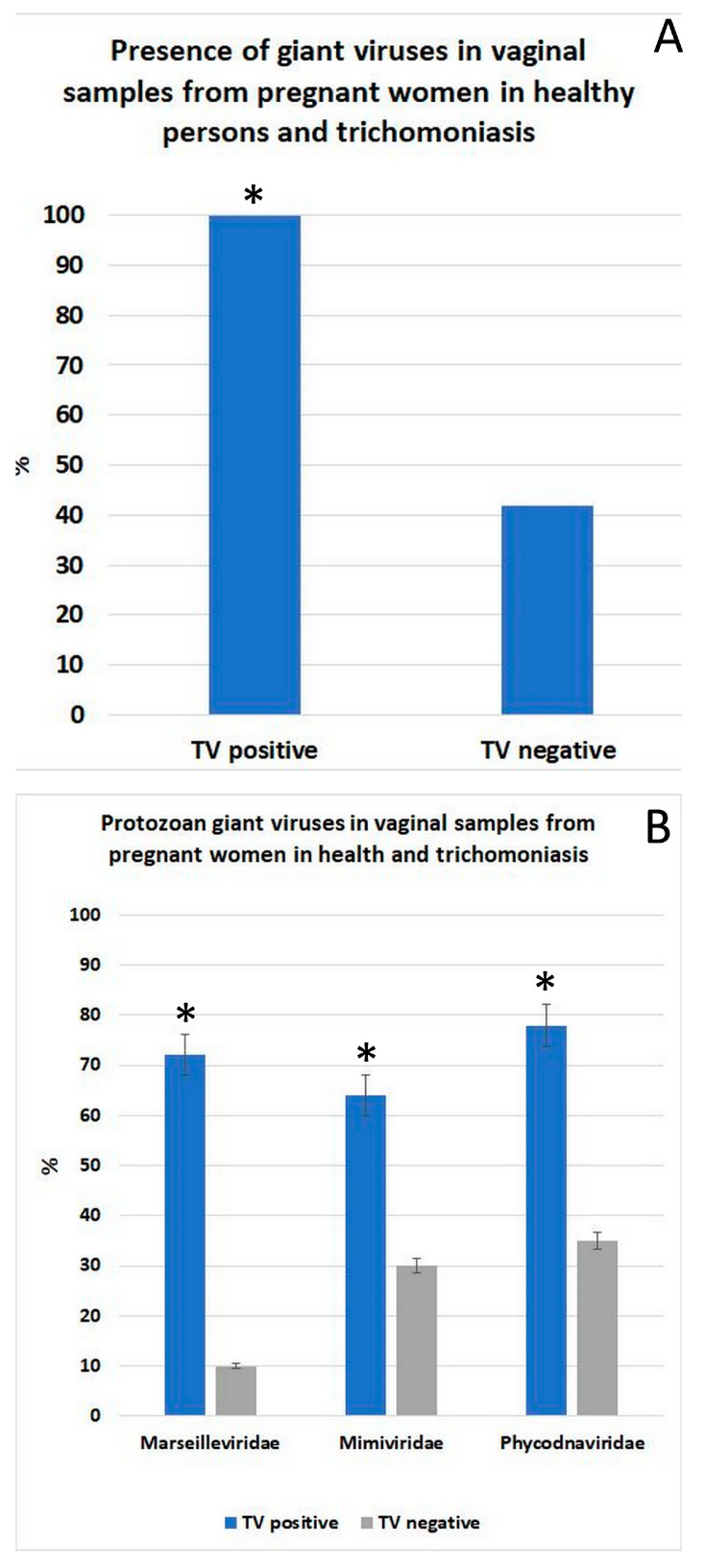
Prevalence of giant viruses in vaginal samples from pregnant women, comparing *Trichomonas vaginalis* (TV)-positive and TV-negative individuals. (**A**) Overall detection rate of giant viruses in vaginal samples shows significantly higher prevalence in TV-positive individuals compared with TV-negative individuals (*p* < 0.05). (**B**) Distribution of giant viruses by viral family (*Marseilleviridae*, *Mimiviridae*, and *Phycodnaviridae*) in TV-positive (blue bars) and TV-negative (gray bars) samples. * TV-positive samples showed significantly higher detection rates for all three viral families (*p* < 0.05–*p* < 0.01).

We examined the prevalence of giant protozoan virus species in vaginal samples collected from women with TV infection (n = 32) and from healthy pregnant women (n = 30). All women with trichomoniasis were found to carry various giant protozoan viruses in their vaginal samples, whereas such viruses were detected in approximately 40% of healthy pregnant women (Figure 5A). The most frequently identified viruses belonged to the *Phycodnaviridae* family, followed by members of the *Marseilleviridae* and *Mimiviridae* families (Figure 5B).

### 3.6. Transcriptional Activity of Giant Virus Genes in TV Cultures

As shown in Figure 3, there was no statistically significant increase in the number of giant protozoan viruses in TV cultures after 48 h of incubation.

**Figure 6:**
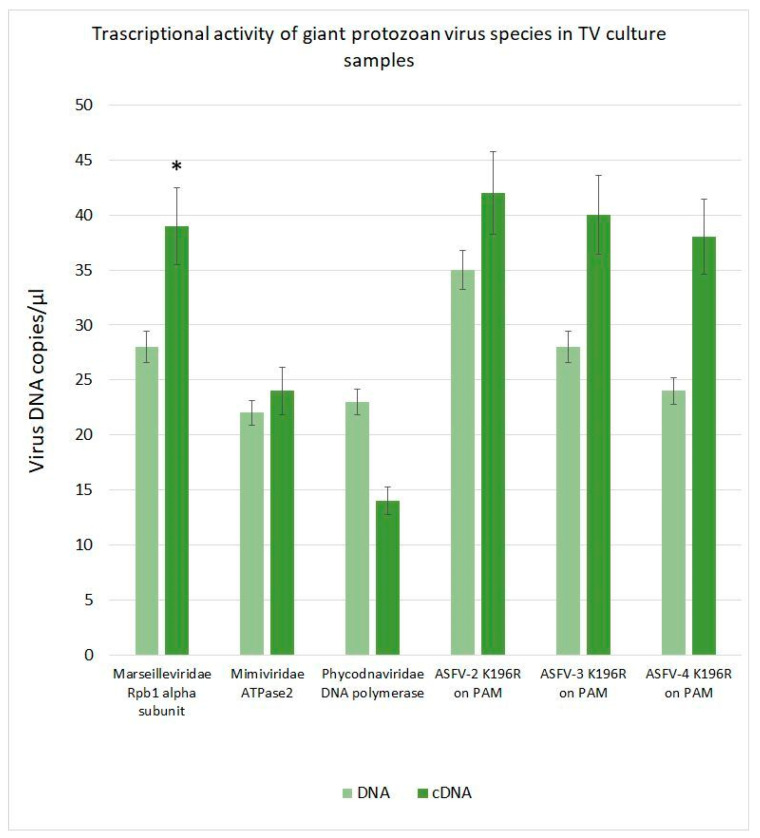
The transcriptional activity of giant protozoan virus genes in TV cultures. * significant compared with *Mimiviridae* and *Phycodnaviridae.* A comparison with the transcriptional activity of African swine fever virus genes during in vitro infection is shown.

The transcriptional activity of giant protozoan virus genes in TV cultures is presented in Figure 6. After 48 h of cultivation, only viruses from the *Marseilleviridae* family exhibited detectable transcriptional activity, specifically in the Rpb1 alpha subunit gene. In contrast, no transcription was observed for the DNA polymerase gene of *Phycodnaviridae* family viruses. The transcriptional data for *Mimiviridae* family viruses, based on *ATPase* gene expression, were contradictory.

## 4. Discussion

TV is associated with considerable discomfort and has been linked to adverse pregnancy outcomes. However, certain aspects of these pathologies remain underexplored. Infection with the novel TV strain is most likely transmitted vertically, although horizontal transmission cannot be entirely ruled out. It is well known that treatment for trichomoniasis during pregnancy may also pose risks. The co-infection hypothesis is emphasized in the literature, with several studies suggesting that most TV infections in pregnant women occur alongside other microbial infections. In the immunocompromised state of pregnancy, these co-infections may potentially lead to complications for both the mother and the unborn child [21,22,23].

In recent decades, there has been growing attention on TVV infections caused by dsRNA viruses, as these viruses are believed to influence trichomonad virulence and pathogenesis [24,25,26]. It is currently understood that five types of viruses can exist in various combinations within a single Trichomonas organism. Our data provide information on the prevalence of TVV1 to TVV5 strains in pregnant women in Armenia.

It is important to note that the prevalence of TV among pregnant women is less than 1%, which makes it somewhat challenging to collect samples for studying TVVs. However, according to the statistical data we received, all TVV strains were present in TV, with TVV3 and TVV4 being the most prevalent. Literature data indicate that the most common strain is TVV1, followed by TVV2 and TVV3. The role of these strains in human pathogenesis has been more extensively explored [21,22,27]. The combination of TVV strains varies depending on TV isolates and demographics. It is well documented that TVV4 was first isolated from Italian TV strains in 2019, marking its first detection in Europe. Moreover, Australian TV isolates were found to lack TVV4, and any TVVs were harbored in TV *actin* genotype E from pregnant women in Kilifi, Kenya [18,28,29]. The number of TVVs in vaginal samples is directly linked to the number of protozoa, as the virus can only replicate within the TV. Similar results were observed when protozoa were cultured in vitro. In the studied samples, a quantitative increase in the virus types was observed in comparison with vaginal smear samples. Based on this information, it is possible to indirectly estimate the amount of TV in patients.

The association between TVVs and human pathology remains highly controversial in the literature. Some studies suggest that TVVs are not linked to adverse pregnancy outcomes, while others report a potential association [18,23,24,30,31,32,33]. A new representative of TVV was only identified in 2022 [11], raising the possibility that earlier studies may have overlooked certain viral strains. Consequently, cases previously reported as TVV-negative in TV-positive patients may, in fact, have involved undetected viral co-infections. In addition to the pathological effects attributed to TV, pregnancy complications may also be influenced by social determinants of health. Based on clinical interviews, no overt socioeconomic stressors, such as inadequate housing, poor nutrition, or domestic violence, were reported among the pregnant women evaluated. However, these factors were not systematically assessed and therefore cannot be ruled out.

Our findings are consistent with previous research [4] suggesting that two key complications—preterm birth and premature rupture of the amniotic membrane—may be associated with either TV or TVV. However, it is challenging to attribute these outcomes solely to TV or TVV, as all identified cases of TV infection were accompanied by viral presence. Confirmation of these observations requires further investigation.

It is reported that high vaginal viral diversity has been linked to preterm birth [30]. All three of the most common families of giant protozoan viruses examined in our study have been identified in the vaginal virome [1,31,32,33].

One of the most important unresolved questions is how these giant protozoan viruses are able to infect the female genital tract. While the direct association between TVV and TV is well established, there is currently no consensus regarding the role or mechanism of infection of giant protozoan viruses in this context. The presence of giant viruses in the female genital tract may result from several potential routes of transmission, including sexual intercourse, contamination from the fecal microbiota, exposure to contaminated water (e.g., swimming), and possibly hematogenous spread of certain viruses, such as that of *Marseillevirus*. In 2012, a study reported the detection of *Marseillevirus*-related sequences in a stool sample from a healthy Senegalese man. These findings were confirmed through viral culture, representing the first identification of a giant virus in the human gut [34]. Subsequent research has demonstrated that sequences related to the *Marseilleviridae* family are frequently present in human fecal samples, including those from individuals with inflammatory bowel disease as well as from healthy controls [35]. These findings suggest that *Marseilleviridae-like* viruses may be a component of the normal human gut virome; however, their precise role in human health and disease remains unclear. *Mimiviruses* have been detected in both human intestinal and respiratory samples, indicating their presence within the human body. In 2012, a strain of *Mimivirus* was isolated from the stool of a Tunisian girl with pneumonia, marking the first documented isolation of a *Mimivirus* from human feces. This finding suggests that these viruses can inhabit the human gastrointestinal tract [36]. However, subsequent studies have shown that, while Mimivirus-like sequences are detectable in stool samples, their presence does not necessarily correlate with gastrointestinal disease. Their detection may be incidental, potentially reflecting environmental exposure or association with free-living amoebae rather than a direct pathogenic role [34].

Metagenomic studies have identified sequences related to *Phycodnaviridae* in human fecal samples. For instance, a study analyzing the gut virome of individuals from the Indian subcontinent found that members of the *Phycodnaviridae* family were present in all analyzed individuals, suggesting their existence as part of the human gut virome [37,38]. The presence of *Phycodnaviridae* in the human gut may result from the consumption of contaminated food or water, as these viruses are known to infect algae and can be transmitted through environmental sources. The detection of giant viruses in vaginal samples raises important questions about their origin and role in the urogenital tract. The presence of these viruses may reflect either transient contamination (e.g., fecal–vaginal transmission) or true colonization. If we exclude technical errors when taking a smear from the vagina, the presence of giant viruses in the vagina may be associated with non-observance of personal hygiene rules. Due to the anatomical features of the female body (close location of the anus and the entrance to the vagina), infection occurs more easily. Muscle relaxation can be caused by pregnancy hormones (progesterone has a muscle relaxant effect), which can be associated with the anal sphincter and contribute to infection [39]. However, due to the nature of our study design, we were not able to distinguish between these possibilities. Further studies are needed to clarify whether these viruses establish persistent infections or represent incidental findings related to environmental exposure or host microbiota dynamic.

The expression of genes from giant viruses of the *Marseilleviridae* family in TV cultures is challenging to explain. One possible hypothesis is that certain giant protozoan viruses may replicate within flagellated protists. Notably, TV can adopt an amoeboid form, which may facilitate viral entry into the cell [40]. However, there is currently no direct evidence supporting the replication of giant protozoan viruses in TV. These assumptions require additional studies. The primary culture of TV is not sterile and contains many microorganisms, so an antibiotic is added to the medium, though absolute sterility is difficult to achieve [15]. Thus, an alternative explanation is that the expression of viral genes may result from the infection of other organisms present in the culture, such as fungi. Yet, to date, there is also no conclusive evidence demonstrating the replication of similar giant viruses in parasitic fungi.

We can assume that the presence of TV is most likely associated with an increased vaginal virome, and that, when treating this disease, the possible response of both changes in the virome and vaginal immunity should be assessed.

## 5. Conclusions

As all TV infections in this study were associated with the presence of TVV1–TVV5, further research is required to elucidate the specific combinations of TVVs present in Armenian isolates and their potential roles in the pathogenesis of trichomoniasis-related complications.

Our findings showed a potential association between TV infection and the presence of giant protozoan viruses. However, the underlying cause of this association remains unclear. It has yet to be determined whether this connection results from sexual transmission, co-infection with TV, or fecal contamination.

## Data Availability

Data is contained within the article or supplementary material.

## Supplementary Materials

The following supporting information can be downloaded at https://www.mdpi.com/article/10.3390/pathogens14080764/s1, Table S1: (Primers used for Trichomonas vaginalis virus and giant viruses determination); Table S2: (Primary clinical and laboratory material); Table S3: (Frequency of pregnancy outcomes in norm and in Trichomoniasis patients in the Republican Institute of Reproductive Health, Perinatology, Obstetrics and Gynaecology in Yerevan).

## Abbreviations

Abbreviation	Full term
hMPV	Human metapneumovirus
RT-PCR	Reverse transcription polymerase chain reaction
CT	Computed tomography
PMCT	Postmortem computed tomography
ED	Emergency department
ICU	Intensive care unit
DM	Diabetes mellitus
HTN	Hypertension
COPD	Chronic obstructive pulmonary disease
GI	Gastrointestinal
CNS	Central nervous system
DAD	Diffuse alveolar damage
LRTI	Lower respiratory tract infection
CRP	C-reactive protein
ALT	Alanine aminotransferase
AST	Aspartate aminotransferase
ALP	Alkaline phosphatase
LDH	Lactate dehydrogenase
GGT	Gamma-glutamyl transferase
H&E	Hematoxylin and eosin
IHC	Immunohistochemistry
DNA	Deoxyribonucleic acid
RNA	Ribonucleic acid

## References

[1] Jakobsen R.R., Haahr T., Humaidan P., Jensen J.S., Kot W.P., Castro-Mejia J.L., Deng L., Leser T.D., Nielsen D.S. (2020). Characterization of the Vaginal DNA Virome in Health and Dysbiosis. Viruses.

[2] Britto A.M.A., Siqueira J.D., Curty G., Goes L.R., Policarpo C., Meyrelles A.R., Furtado Y., Almeida G., Giannini A.L.M., Machado E.S. (2023). Microbiome analysis of Brazilian women cervix reveals specific bacterial abundance correlation to RIG-like receptor gene expression. Front. Immunol..

[3] Rowley J., Vander Hoorn S., Korenromp E., Low N., Unemo M., Abu-Raddad L.J., Chico R.M., Smolak A., Newman L., Gottlieb S. (2019). Chlamydia, gonorrhoea, trichomoniasis and syphilis: Global prevalence and incidence estimates, 2016. Bull. World Health Organ..

[4] Van Gerwen O.T., Opsteen S.A., Graves K.J., Muzny C.A. (2023). Trichomoniasis. Infect. Dis. Clin. N. Am..

[5] Menezes C.B., Frasson A.P., Tasca T. (2016). Trichomoniasis—Are we giving the deserved attention to the most common non-viral sexually transmitted disease worldwide?. Microb. Cell.

[6] Swygard H., Seña A.C., Hobbs M.M., Cohen M.S. (2004). Trichomoniasis: Clinical manifestations, diagnosis and management. Sex. Transm. Infect..

[7] Masha S.C., Cools P., Sanders E.J., Vaneechoutte M., Crucitti T. (2019). Trichomonas vaginalis and HIV infection acquisition: A systematic review and meta-analysis. Sex. Transm. Infect..

[8] Dessì D., Margarita V., Cocco A.R., Marongiu A., Fiori P.L., Rappelli P. (2019). Trichomonas vaginalis and Mycoplasma hominis: New tales of two old friends. Parasitology.

[9] Goodman R.P., Freret T.S., Kula T., Geller A.M., Talkington M.W., Tang-Fernandez V., Suciu O., Demidenko A.A., Ghabrial S.A., Beach D.H. (2011). Clinical isolates of Trichomonas vaginalis concurrently infected by strains of up to four Trichomonasvirus species (Family Totiviridae). J. Virol..

[10] Fraga J., Rojas L., Sariego I., Fernández-Calienes A. (2011). Double-stranded RNA viral infection of Trichomonas vaginalis and correlation with genetic polymorphism of isolates. Exp. Parasitol..

[11] Manny A.R., Hetzel C.A., Mizani A., Nibert M.L. (2022). Discovery of a Novel Species of Trichomonasvirus in the Human Parasite Trichomonas vaginalis Using Transcriptome Mining. Viruses.

[12] Ding H., Gong P., Yang J., Li J., Li H., Zhang G., Zhang X. (2017). Differential Protein Expressions in Virus-Infected and Uninfected Trichomonas vaginalis. Korean J. Parasitol..

[13] El-Gayar E.K., Mokhtar A.B., Hassan W.A. (2016). Molecular characterization of double-stranded RNA virus in Trichomonas vaginalis Egyptian isolates and its association with pathogenicity. Parasitol. Res..

[14] Lu X., Lu Q., Zhu R., Sun M., Chen H., Ge Z., Jiang Y., Wang Z., Zhang L., Zhang W. (2025). Metagenomic analysis reveals the diversity of the vaginal virome and its association with vaginitis. Front. Cell. Infect. Microbiol..

[15] Gelbart S.M., Thomason J.L., Osypowski P.J., Kellett A.V., James J.A., Broekhuizen F.F. (1990). Growth of Trichomonas vaginalis in commercial culture media. J. Clin. Microbiol..

[16] Karalyan Z.A., Ghonyan S.A., Poghosyan D.A., Hakobyan L.H., Avagyan H.R., Avetisyan A.S., Abroyan L.O., Poghosyan A.A., Hakobyan S.A., Manukyan G.P. (2024). Infection of Human Macrophage-Like Cells by African Swine Fever Virus. Front. Biosci..

[17] Yin J.L., Shackel N.A., Zekry A., McGuinness P.H., Richards C., Van Der Putten K., Mccaughan G., Eris J.M., Bishop G.A. (2001). Real-time reverse transcriptase-polymerase chain reaction (RT-PCR) for measurement of cytokine and growth factor mRNA expression with fluorogenic probes or SYBR Green I. Immunol. Cell Biol..

[18] Jehee I., van der Veer C., Himschoot M., Hermans M., Bruisten S. (2017). Direct detection of Trichomonas vaginalis virus in Trichomonas vaginalis positive clinical samples from the Netherlands. J. Virol. Methods.

[19] Avagyan H.R., Hakobyan S.A., Poghosyan A.A., Bayramyan N.V., Arzumanyan H.H., Abroyan L.O., Avetisyan A.S., Hakobyan L.A., Karalova E.M., Karalyan Z.A. (2022). African Swine Fever Virus Manipulates the Cell Cycle of G0-Infected Cells to Access Cellular Nucleotides. Viruses.

[20] Anderson N.G., Waters D.A., Nunley C.E., Gibson R.F., Schilling R.M., Denny E.C., Cline G., Babelay E., Perardi T.E. (1969). K-Series centrifuges I. Development of the K-II continuous-sample-flow-with-banding centrifuge system for vaccine purification. Anal. Biochem..

[21] Allsworth J.E., Ratner J.A., Peipert J.F. (2009). Trichomoniasis and other sexually transmitted infections: Results from the 2001–2004 National Health and Nutrition Examination Surveys. Sex. Transm. Dis..

[22] Asmah R.H., Blankson H.N.A., Seanefu K.A., Obeng-Nkrumah N., Awuah-Mensah G., Cham M., Ayeh-Kumi P.F. (2017). Trichomoniasis and associated co-infections of the genital tract among pregnant women presenting at two hospitals in Ghana. BMC Womens Health.

[23] Hirt R.P. (2013). Trichomonas vaginalis virulence factors: An integrative overview. Sex. Transm. Infect..

[24] Graves K.J., Ghosh A.P., Kissinger P.J., Muzny C.A. (2019). Trichomonas vaginalis virus: A review of the literature. Int. J. STD AIDS.

[25] Khanaliha K., Masoumi-Asl H., Bokharaei-Salim F., Tabatabaei A., Naghdalipoor M. (2017). Double-stranded RNA viral infection of Trichomonas vaginalis (TVV1) in Iranian isolates. Microb. Pathog..

[26] Bahadory S., Aminizadeh S., Taghipour A., Bokharaei-Salim F., Khanaliha K., Razizadeh M.H., Soleimani A., Beikzadeh L., Khatami A. (2021). A systematic review and meta-analysis on the global status of Trichomonas vaginalis virus in Trichomonas vaginalis. Microb. Pathog..

[27] Cotch M.F., Pastorek J.G., Nugent R.P., Hillier S.L., Gibbs R.S., Martin D.H., Eschenbach D.A., Edelman R., Carey C.J., Regan J.A. (1997). Trichomonas vaginalis associated with low birth weight and preterm delivery. The Vaginal Infections and Prematurity Study Group. Sex. Transm. Dis..

[28] Margarita V., Marongiu A., Diaz N., Dessì D., Fiori P.L., Rappelli P. (2019). Prevalence of double-stranded RNA virus in Trichomonas vaginalis isolated in Italy and association with the symbiont Mycoplasma hominis. Parasitol. Res..

[29] Masha S.C., Cools P., Crucitti T., Sanders E.J., Vaneechoutte M. (2017). Molecular typing of Trichomonas vaginalis isolates by actin gene sequence analysis and carriage of *T. vaginalis* viruses. Parasites Vectors.

[30] Stout M.J., Brar A.K., Herter B.N., Rankin A., Wylie K.M. (2023). The plasma virome in longitudinal samples from pregnant patients. Front. Cell. Infect. Microbiol..

[31] Happel A.U., Varsani A., Balle C., Passmore J.A., Jaspan H. (2020). The Vaginal Virome-Balancing Female Genital Tract Bacteriome, Mucosal Immunity, and Sexual and Reproductive Health Outcomes?. Viruses.

[32] Wylie K.M., Wylie T.N., Cahill A.G., Macones G.A., Tuuli M.G., Stout M.J. (2018). The vaginal eukaryotic DNA virome and preterm birth. Am. J. Obs. Gynecol..

[33] Li F., Chen C., Wei W., Wang Z., Dai J., Hao L., Song L., Zhang X., Zeng L., Du H. (2018). The metagenome of the female upper reproductive tract. Gigascience.

[34] Colson P., Fancello L., Gimenez G., Armougom F., Desnues C., Fournous G., Yoosuf N., Million M., La Scola B., Raoult D. (2013). Evidence of the megavirome in humans. J. Clin. Virol..

[35] Zuo T., Lu X.J., Zhang Y., Cheung C.P., Lam S., Zhang F., Tang W., Ching J.Y.L., Zhao R., Chan P.K.S. (2019). Gut mucosal virome alterations in ulcerative colitis. Gut.

[36] Saadi H., Reteno D.G., Colson P., Aherfi S., Minodier P., Pagnier I., Raoult D., La Scola B. (2013). Shan virus: A new mimivirus isolated from the stool of a Tunisian patient with pneumonia. Intervirology.

[37] Ansari M.H., Ebrahimi M., Fattahi M.R., Gardner M.G., Safarpour A.R., Faghihi M.A., Lankarani K.B. (2020). Viral metagenomic analysis of fecal samples reveals an enteric virome signature in irritable bowel syndrome. BMC Microbiol..

[38] Bhardwaj K., Garg A., Pandey A.D., Sharma H., Kumar M., Vrati S. (2022). Insights into the human gut virome by sampling a population from the Indian subcontinent. J. Gen. Virol..

[39] Sitaraman L., Lewicky-Gaupp C., Rao S.S. (2025). Postpartum Anorectal and Pelvic Floor Disorders: Evaluation, Treatment, and Prevention. Curr. Gastroenterol. Rep..

[40] Arroyo R., González-Robles A., Martínez-Palomo A., Alderete J.F. (1993). Signalling of Trichomonas vaginalis for amoeboid transformation and adhesion synthesis follows cytoadherence. Mol. Microbiol..

