# Supplementary material for: Presence of Protozoan Viruses in Vaginal Samples from Pregnant Women and Their Association with Trichomoniasis
Source: Pathogens. 2025 Aug 1;14(8):764. doi: 10.3390/pathogens14080764 (PMC12389363; doi:10.3390/pathogens14080764)
Supplement: Supplementary file 1 [file pathogens-14-00764-s001.zip › Table S1.pdf]

Table S1. Primers used for Trichomonas vaginalis virus and giant viruses determination

TVV-1	F-CGATTACAGTTCATTCAC, R-CGTCATTACATAAGTCTTG, F-CAAGATTATTAGCGGTGTT, R-GACGGAATATAGGTGAGTA.
TVV-2	F-ATTCTCGCTGATGGTATAG, R-CCGTTGAACAAGTAATCG, F-ATCGTCTGATAATTCTGCCAC, R-GTGTCGGGAGGTGAAGTA
TVV-3	F-ATACTGAAGTACGCTCACCTG, R-GTTTGACACACGGCTTTGA, F-ATACTGAAGTACGCTCACCTGAT, R-TTTGACACACGGCTTTGAGT
TVV-4	F-ACTATTTCTTCTGCTAATCTT, R-AATGTGGTGAATCTGTTG, F-TATTTCTTCTGCTAATCTTAATG, R-AATGTGGTGAATCTGTTG
TVV-5	F-TCCACTATGTAGAAGAGAA, R-AAGAGATGCTAACCTGTA
Mimivurus R651	F-CAGAATCAATCACCGAATC R-CCAATAGTATCAACAGAATCAT
Mimivirus BAV	F-CAGATTCTACTTACAGTGTCAATA R-GACCAGTATGTGCTTCAAC
Phycodnavirus LO20	F-GAGTCTGTATCCGAGTATCA R-TTGTCAAACTTATTCTCCATCA
Phycodnavirus LO08	F-GTCATATACGGCGATACTG R-GTGGTCATTGATGTCTTCT
Marselliavirus Rpb1 alpha subunit	F-AGGTTGAACTCGTCTTCTCTT R-AGTATCGCAAGGTGTCTGA
Marselliavirus DNA polymerase	F-GCTTTGAGATTCTCTCTGCTCTCG R-ACACCGTCTGTCTGTCGATGA
