# Supplementary material for: Presence of Protozoan Viruses in Vaginal Samples from Pregnant Women and Their Association with Trichomoniasis
Source: Pathogens. 2025 Aug 1;14(8):764. doi: 10.3390/pathogens14080764 (PMC12389363; doi:10.3390/pathogens14080764)
Supplement: Supplementary file 1 [file pathogens-14-00764-s001.zip › Table S2.pdf]

Table S2. Primary clinical and laboratory material

	TVV combinations	Gardnerella	clue cells	candida spp	cocci	E. coli	syphilis	gonorrhea	HHV-2	abort alert	Vaginal discharge	itch	Colpitis	Dysuria	Colpitis macularis	Premature birth	Premature rupture of membranes	Low Birth Weight	Complicated obstetrical anamnesis	Abortion	Harmful habits	Caesarean section in the history	History of natural childbirth
1	1,3,5	+	+	0	+	0	0	0	0	+	+	+	+	0	0	+	+	+	0	0	0	+	+
2	3	0	+	0	+	0	+	0	0	+	+	+	0	0	0	0	0	+	+	0	0	0	+
3	2,3,4,5	0	0	+	0	0	0	0	0	+	+	+	0	+	0	+	+	+	0	0	0	0	0
4	2,3	+	+	+	0	0	0	0	0	+	+	+	+	0	+	+	0	+	0	0	0	0	+
5	3,4,5	0	+	+	+	+	0	0	0	0	+	+	+	+	0	0	0	0	0	0	0	+	0
6	1,4,5	0	+	0	0	0	0	0	0	+	+	0	+	+	0	0	0	0	+	0	0	+	0
7	3,4,5	0	+	0	0	0	0	+	0	+	+	0	+	0	0	+	+	+	0	0	0	+	0
8	4	+	+	0	0	0	+	0	0	+	+	+	+	0	0	0	0	0	+	+	0	0	0
9	4,5	0	+	0	0	0	0	0	0	+	+	0	+	0	0	0	0	0	0	+	0	0	0
10	1,2,3,4,5	+	+	0	+	+	0	0	+	0	+	+	+	+	+	+	+	+	+	0	0	0	+
11	1,2,3	0	+	0	+	0	0	0	0	+	+	+	+	0	0	+	0	0	+	0	0	0	+
12	1,2,3	+	+	0	0	0	0	+	0	+	+	+	+	0	0	+	+	+	0	0	0	+	0
13	1,2,3,4	0	+	0	0	0	0	0	0	0	+	+	+	0	+	+	+	+	+	0	0	0	0
14	1,2,3,4	0	0	0	+	0	0	0	0	0	+	+	+	0	+	0	0	0	0	0	0	0	+
15	1,2,3	+	+	0	+	0	0	0	0	+	+	+	+	+	0	0	0	0	+	0	0	+	+
16	1,2,3	+	+	+	0	0	0	0	0	+	+	0	0	0	0	0	0	0	0	0	0	+	0
17	1,2,3,4	+	+	0	0	0	0	0	0	+	+	+	+	+	+	+	+	+	0	0	0	+	0

18	2,3,4	+	0	0	+	0	0	0	0	+	+	+	+	+	0	0	0	0	+	+	0	0	+
19	1,2,3,4	+	+	+	0	0	0	0	0	+	+	+	+	+	+	0	0	0	+	0	0	+	0
20	1,2,3,4,5	+	+	+	+	+	0	0	0	+	+	+	+	+	+	+	+	+	+	0	0	0	0
21	1,3,5	+	+	+	+	0	0	0	0	+	+	+	+	0	0	0	0	0	+	0	0	0	0
22	1,3,4	+	+	0	+	0	0	0	0	+	+	0	+	+	0	0	0	0	+	0	0	0	+
23	1,3,5	+	+	+	+	0	0	0	0	+	+	+	+	0	0	0	0	0	0	0	0	0	+
24	1,3,4,5	0	0	0	+	0	0	+	0	+	+	+	+	+	0	0	+	0	+	0	0	+	0
25	1,3,5	+	+	0	+	0	0	0	0	+	+	0	+	0	0	0	0	0	+	0	0	0	0
26	1,3,5	0	0	+	0	0	0	0	0	+	+	+	0	+	0	0	0	0	0	+	0	0	+
27	1,3,4	+	+	0	+	0	0	0	0	+	+	+	+	0	0	0	0	+	0	0	0	0	0
28	1,2,3,4	0	0	+	+	0	0	0	0	+	+	+	+	0	+	0	0	0	0	0	0	0	0
29	1,2,3,4,5	+	+	0	+	+	+	0	0	0	+	+	+	+	+	+	+	+	+	0	0	0	+
30	1,2,3,4,5	+	+	0	+	0	0	0	0	+	+	+	+	+	+	0	0	+	0	0	0	+	+
31	1,3,4,5	+	+	0	+	+	0	0	0	+	+	+	+	0	+	0	+	0	+	0	+	0	+
32	1,2,3,4,5	+	+	+	+	+	0	0	0	+	+	+	+	+	+	+	+	0	0	0	0	0	0
