# Supplementary material for: Presence of Protozoan Viruses in Vaginal Samples from Pregnant Women and Their Association with Trichomoniasis
Source: Pathogens. 2025 Aug 1;14(8):764. doi: 10.3390/pathogens14080764 (PMC12389363; doi:10.3390/pathogens14080764)
Supplement: Supplementary file 1 [file pathogens-14-00764-s001.zip › Table S3.pdf]

Table S3. Frequency of pregnancy outcomes in norm and in Trichomoniasis patients in the Republican Institute of Reproductive Health, Perinatology, Obstetrics and Gynaecology in Yerevan. Values are numbers (percentages).

Pregnancies	Pregnancy loss			Live births		Ongoing pregnancy
	Miscarriage		Stillbirth	1764 (50.74%)		
	309 (8.9%)					
	Spontaneous abortion	Induced abortion		Term birth	Preterm birth and preterm premature rupture of membranes (PPROM)	
3476	278 (7.99%)	31 (0.9%)	92 (2.6%)	1681 (48.30%)	83 (2.3%)	1311 (37.7%)
Trichomoniasis						
32	4 (12.5%)	0 (%)	1 (3.12%)	5 (15.62%)	12 (37.5%)*	10 (31.25%)

*Chi-square test: value 148.6; df 1.
